# The Relation of Inspectors to Infirmary Superintendents

**Published:** 1913-04-12

**Authors:** 


### THE RELATION OF INSPECTORS TO INFIRMARY SUPERINTENDENTS.

### Dr. E. E. Norton, Brentford Union Infirmary, gives his Views.

State inspection of any kind, and the word inspection itself, is not pleasing to most institutional workers, for whom it carries, in the abstract at least, an atmosphere of interference, and perhaps of want of sympathy. But, on the other hand, those classes of institutions for which State inspection and the visits of inspectors are a matter of routine are known to hold much less dubious views of the matter. As there is still some confusion of thought on the subject, and the institutions which are not subject to inspection at present generally retort to suggestions for its introduction : "We at least have nothing to fear," as if that were one that could be said in its favour, the value of the inspector to the Poor-Law infirmary, in the light of experience, becomes a question full of suggestion to other institutions, apart from its intrinsic interest. In order to complete the summary of infirmary matters contained in last week's Special Number of The Hospital, therefore, our representative visited Dr. E. E. Norton, the Medical Superintendent of Brentford Union Infirmary, Isleworth, to ascertain his views.

### Changes in the Outlook of Inspectors.

"Great developments have certainly attended the Poor-Law infirmary of late years," began Dr. Norton, "and as a part of this progress, which may be summarised as the further emerging of the infirmary from the workhouse ward into a separate self-contained institution, there have been changes in the outlook of inspectors, as in that of other officials. To go no further back than the early 'nineties, let me give you an instance of the change out of my own experience. At that time the inspectors, or certainly the older among them, belonged to a school which insisted that destitution must always take precedence of every other claim for admission to the sick wards, as to all others in the workhouse. So, though there existed what had come to be known as the infirmary, I well remember inspectors insisting that every patient's bed-card should bear the word 'Workhouse' upon it, and complaining to the medical officer if this were not done. This, of course, is not the case now. In those days, too, the old idea of an inspector, as a visitor who came to spy out the land, had something of reality since complaints were much more frequent; and I believe I am right in saying that to-day the inspectors have far more trouble with unseparated infirmaries."

"Are their duties and visits such as to assist the medical superintendents?"

"Their visits are of the greatest value, and though the scope of their duties is carefully defined by their knowledge of the Infirmary Order, and they are guided by the three main sources of information—the reports of the Guardians, press cuttings, and letters and interviews—they are able by these means to help very greatly. Indeed, it would be more true to speak of the co-operation than the inspection of inspectors. Their function, at least from the medical superintendent's point of view, is to provide a channel of intercommunication, so that we may know the result of their experiences elsewhere. A medical superintendent of an infirmary cannot always know what experiments and solutions have been carried out in other infirmaries. The inspector provides a link of progress between one institution and another. It is obvious, on reflection, that the inspector coming from outside can notice things which the superintendent, who is engaged with looking after the sick, might not see. Then the inspectors will always answer letters, and will come down by request for a special purpose."

### The Rareness of Medical Inspections.

"Are infirmaries over-inspected?"

"Far from it. In my opinion there is a tendency to under-inspection. What I mean is this : A superintendent sometimes regrets that the medical inspections have become, except for special reasons, less frequent. The reason appears to be that the medical inspectors are so few in number that they simply cannot get round all the institutions as a matter of routine. There are Sir Arthur Downes and Dr. Fuller, but it is impossible for two men to undertake routine inspection when there are so many claims on their special experience to meet. Thus a medical superintendent, who is really proud of his institution, at times feels the lack of the approval of some one who is technically competent to judge. Boards of Guardians, however valuable their co-operation, must necessarily take an insular view, for they have not the means of comparison, which are the most valuable asset of the inspector. The lay inspectors are far more numerous, and their visits are highly valuable. Should a superintendent happen to be out at the time of the visit, he will greatly regret the lost opportunity of a conversation with the inspector. But if he feels this, as I certainly do, in regard to the lay inspector, it is clear that his regret is the keener that medical inspections are not more frequent. At the same time, the wide experience and knowledge of all the inspectors and the invaluable assistance of the lady inspectors in matters of nursing and the care of children have had a beneficial effect upon infirmary management which cannot be over-estimated."

"What will be the future of medical inspection?"

"Forecasts are always difficult—and forecasts about the future of the Poor Law at the present time unusually so. There are signs of change, and no one can yet say which of the proposed changes will take place. In this sense the Poor Law is marking time at present. It is in the melting-pot, and fresh developments for the moment are arrested. Boards of Guardians think that the effect of old-age pensions, of the Insurance Act, of possible legislation for the feeble-minded may be to absorb Poor-Law work into a new administration. You see, it is possible that either of the two Reports of the Royal Commission on the Poor Law will be wholly or in part adopted, and whichever be adopted the effect will be the re-casting of Poor-Law control. Whether Boards of Guardians will survive, or whether committees of the County Councils will be made responsible for their work, are questions outside the scope of Poor-Law inspection, but I mention their existence to show how difficult it is to forecast the future development of the infirmary or the inspector."

### Infirmaries and Tuberculosis.

"In the question of tuberculosis, development, however, is being forced on?"

"It is true that the infirmaries may be in time freed from tuberculosis work. It is felt by many authorities that infirmaries do not provide sufficient scope for the classification of selected cases, with a better chance of recovery, or for their supervision after their discharge from the phthisical wards. The inspectors are satisfied that there is enough accommodation for advanced but not for incipient cases, and the public measures now being taken for the control of the disease are tending, naturally, to throw on every institution, including the infirmary, patients in earlier stages than was formerly the case. This question is simply an illustration of the inspector's point of view as regards the possibilities of the infirmary at the present time.

A Link in the Court of Appeal.

"There is, of course, the administrative side of the inspector's work, though that is more related to the Guardians than to the medical officer. The inspector is a link in the court of appeal, to whom the aggrieved officer or patient may appeal. But the inspector, which is a way of showing how much the office has developed from its early functions, will interfere only when advice is asked for. For example, he now seldom discusses individual medical treatment. His guide is the Infirmary Order, and he inquires why in one section more is spent by one infirmary than by another. He is concerned to prevent overcrowding, and to secure proper allocation for infectious diseases. On questions of expenditure his advice is always asked. In The Hospital lately the question of septic theatres for infirmaries has been once more brought to light. It may serve as an example of the sort of question on which an inspector is consulted. He would admit the medical superintendent's contention that there is a larger proportion of septic cases in infirmaries, because the voluntary hospitals evade as far as they can the admission of such cases for fear of the risk of spreading infection. Nobody wants them, but the infirmaries are virtually bound to accept them. As far as I know there are no infirmaries in London with one theatre for septic and another for non-septic cases. The inspector, therefore, consulted on such a point by a medical superintendent who desired a theatre for septic cases, would be in sympathy with the peculiar circumstances which the latter might urge, however considerations of expense and so on might lead him eventually to advise the Guardians. Moreover, however the matter were decided, the medical superintendent would feel that his case had been heard by competent ears, and might hear of some alternative supposing his suggestion were negatived in the end."

Inspectors and Future Legislation.

"Whatever the coming legislation, the inspector will be of value?"

"Yes, for whatever form that legislation may take—and one cannot forecast its details—it will probably take the form of bringing the infirmaries into line with the voluntary hospitals. At present the voluntary hospital is able to some extent to select its cases, because it knows that the infirmary is in the background. But, unfortunately, there is no easy communication between the two, and the line of transfer is a troublesome one. The doctor on the voluntary hospital's staff has to tell the patient's friends that the case is unsuitable for his institution, and that they had better apply to the relieving officer. Now, though a patient who has once had experience of an infirmary is always ready to return in case of need, the theoretical qualification of destitution still exists. In practice, every sick person who comes to Isleworth is taken straight into the infirmary, and then, if unsuitable, is taken to the workhouse afterwards. But the relieving officer must first be communicated with. This entails delay; and thus arise difficulties—for time is often short, while the case is urgent—in the transfer of patients from voluntary hospitals to infirmaries. Patients who are not so transferred have set up a simpler procedure.

The Public and the Guardians.

"Large numbers apply at the gate for admission, and are admitted; for the public will not stand the refusal of any applicant by a rate-supported institution. The Guardians, however, can recover fees at law, and do their best to exact a reasonable sum from patients who are received but are not destitute. I have known cases received from which as much as £5 has been recovered for a fortnight's treatment, so far from poor may be the patients infirmaries are asked to admit. In this matter of receiving patients the public have succeeded in over-riding the legal position of the Guardians. Here, again, is an example of the position which made me say that the Poor Law is in the melting-pot at the present time. When the issue is decided by Parliament, the inspectors should be of the greatest use in guiding legislation, for they are in the position of consultants to the infirmaries at large. It is here, too, one is inclined to regret the small numbers of the medical inspectors and the limited opportunities for interchange of views. Not only are the inspectors not antagonistic, but they have a unique opportunity for getting things done for the benefit of the work. Their relation to the medical superintendents is that of experienced consultants. The lay inspectors are entitled by their numbers to be called the primary inspectors; the medical inspectors have become rather a court of appeal on medical questions. And whatever things the modern infirmary suffers from, over-inspection is not one of them."

